# Microbiologic Profiles of Patients with Dental Prosthetic Treatment and Periodontitis before and after Photoactivation Therapy—Randomized Clinical Trial

**DOI:** 10.3390/microorganisms9040713

**Published:** 2021-03-30

**Authors:** Raluca Cristina Mocanu, Maria-Alexandra Martu, Ionut Luchian, Irina Georgeta Sufaru, George Alexandru Maftei, Nicoleta Ioanid, Silvia Martu, Monica Tatarciuc

**Affiliations:** 1 Faculty of Dentistry, “Grigore T. Popa” University of Medicine and Pharmacy, 16 Universitatii Str., 700115 Iasi, Romania; ralucamoc@yahoo.com; 2 Department of Periodontology, “Grigore T. Popa” University of Medicine and Pharmacy, 16 Universitatii Str., 700115 Iasi, Romania; irina_ursarescu@yahoo.com (I.G.S.); parodontologie1@yahoo.com (S.M.); 3 Department of Oral Medicine, “Grigore T. Popa” University of Medicine and Pharmacy, 16 Universitatii Str., 700115 Iasi, Romania; george-alexandru.maftei@umfiasi.ro; 4 Department of Dental Prosthetics, “Grigore T. Popa” University of Medicine and Pharmacy, 16 Universitatii Str., 700115 Iasi, Romania; nicole_ioanid@yahoo.com; 5 Department of Dental Technology, “Grigore T. Popa” University of Medicine and Pharmacy, 16 Universitatii Str., 700115 Iasi, Romania; monica.tatarciuc@umfiasi.ro

**Keywords:** periodontitis, edentation treatment, dental bridges, scaling and root planing, photoactivation therapy, chlorhexidine, bacteria

## Abstract

Fixed prosthodontic dental restorations can potentially affect the periodontal tissues and vice versa, the periodontium can influence the longevity and esthetic appearance of dental restorations. We proposed an investigation on total bacterial load, specific periodontal pathogens, and periodontal clinical parameters in patients with dental fixed prosthesis and different degrees of periodontal tissue loss that followed photoactivation therapy (PDT) adjunctive to scaling and root planing. The study was conducted on 160 subjects, which were randomly assigned to scaling and root planing (SRP) alone (52 subjects, 256 sites), SRP and chlorhexidine rinsing (58 subjects, 276 sites), and SRP plus PDT (50 subjects, 318 sites). Periodontal parameters (plaque index, bleeding on probing, probing depth, and clinical attachment loss), followed by total bacterial load and specific periodontal pathogens (*Aggregatibacter actinomycetemcomitans, Porphyromonas gingivalis, Tannerella forsythia,* and *Treponema denticola*) were examined in each patient at baseline, one and six months after. PDT exerted significant improvements both in clinical and microbiological load after one month, and these results were maintained 6 months after when compared to chlorhexidine rinsing or SRP alone, especially in severe periodontitis cases. Photoactivation therapy as an adjunctive periodontal therapeutic method was efficient in offering supplementary periodontal improvements in the clinical and microbiological parameters of patients with fixed dental prosthesis, particularly in severe periodontitis cases.

## 1. Introduction

Dental biofilm is the main etiological factor for periodontal disease [1], and it develops over a period of several weeks, initially with a supragingival onset, with a mature subgingival biofilm that stabilizes up to 12 weeks. As the biofilm accumulates, there is a colonization of several periodontal bacteria (for example, *Aggregatibacter actinomycetemcomitans Fusobacterium nucleatum; Porphyromonas gingivalis; Prevotella intermedia; Treponema denticola*) [2]. Bacterial biofilm will trigger a host immune response with inflammatory characteristics, including activation of leukocytes, neutrophils, and T lymphocytes and release of antibodies, lipopolysaccharides, and chemical inflammatory mediators that include cytokines and chemokines [3]. If periodontopathogenic infection and periodontal inflammation do not resolve, tissue destruction can occur, such as periodontal pockets, loss of periodontal attachment, bleeding, and bone loss, ultimately resulting in tooth loss [4]. A new system for classifying periodontal diseases was proposed at the 2017 World Workshop, with a system of stages and degrees for the severity of periodontitis [5].

Therapeutic procedures such as scaling and root planning (SRP) may cause a temporary decrease in the level of subgingival bacteria, but it may not completely eliminate periodontopathogenic microorganisms. The localization of these bacteria in hard-to-reach areas, such as the furcation area or the base of the periodontal pockets, is likely to be a failure of mechanical therapy [6]. To this end, various forms of adjunctive periodontal therapies have been developed. Nevertheless, it has been shown that systemic administration of antibiotics can exert some negative side effects, including antibiotic resistance. Local forms of antimicrobial application also have a number of disadvantages, such as the need to repeat treatments, the effect of rapid washing of the substance and, in addition, demineralization of the root surface [7,8]. Another harmful consequence is that SRP can open dentinal tubules, which can further offer access to periodontal bacteria.

A proposed form of non-invasive adjunctive periodontal therapy is photodynamic antibacterial therapy (PDT), which uses a photosensitizing substance and a light source (laser or LED - light emitting diode). This type of therapy generates damage to various components of microbial cells or can irreversibly alter metabolic activity, leading to microbial elimination. The mechanism of action is based on the energy absorbed by intracellular photosensitization, which is transferred to the oxygen molecule to influence the oxidative reaction pathways in the plasma membrane and the genetic material of microbial cells [9]. The efficiency and reliability of PDT is due to the relatively simple basic principles behind it. If the therapeutic steps are followed and all PDT components (light and photosensitizing substance) are present in sufficient quantities during the application of this therapy, then this technique can be extremely effective [10].

The advantages of PDT compared to other treatments are that the photosensitizer can be inserted directly into the periodontal pocket and then activated with a fiber optic tip. Another important advantage is that PDT is only effective against microbial cells, avoiding damage to host tissues; thus, this procedure is safe [11].

There are data from the literature that support potential improvements after using a PDT together with SRP [12]; however, there is some research that reports no additional benefit [13]. Another study [14] demonstrated that the combination of conventional mechanical periodontal treatment with PDT provides supplementary benefits by reducing probing depth and increasing the level of periodontal clinical attachment.

To our knowledge, there are no data on the potential effects of photodynamic therapy on patients with fixed dental prosthesis and periodontal damage of different severity stages. We also did not identify research that reported the beneficial effects of photodynamic therapy in such patients to the so-called “gold standard” in periodontal antibacterial therapy: chlorhexidine. The null hypothesis in this case is that adjunctive photodynamic therapy offers no additional benefits to patients with periodontitis and fixed dental prosthesis when compared to scaling and root planning alone in terms of clinical and microbiological examination.

Therefore, we propose a study to evaluate the efficacy on clinical periodontal parameters and periodontal pathogenic microorganisms of photoactivation therapy and irrigations with chlorhexidine 0.2% solution, which is supplementary to etiological periodontal treatment in patients with conventional fixed prosthetic treatment with periodontal tissue breakdown.

## 2. Materials and Methods

### 2.1. Trial Design

The present study was a randomized single-center clinical trial performed on a group of 169 patients with conventional fixed prosthetic treatments on natural teeth. 

### 2.2. Study Group

The study was performed on subjects addressing The Dental Unit of The University of Medicine and Pharmacy “Grigore T. Popa” Iasi, Romania between 1 October 2017 and 1 October 2019. The inclusion criteria were systemically healthy patients over the age of 18 with a fixed dental prosthetic metal–ceramic bridge exclusively on natural teeth, which included at least 2 abutments and with periodontal disease, with at least 10 remaining teeth in the oral cavity. The exclusion criteria from the study were uncontrolled systemic medical conditions, diabetes or other metabolic disorders, autoimmune diseases, rheumatic diseases, smoking, use of systemic antibiotics and anti-inflammatory drugs or periodontal therapy in the last six months prior to the study.

Prior to the start of the study, all participants were informed about the study and signed a written agreement. The methodology of the study complied with the rules set out in the Helsinki Declaration. The study protocol was approved by the Ethics committee of The University of Medicine and Pharmacy “Grigore T. Popa” Iasi, nr. 19 July 2017.

### 2.3. Sample Size

Power analysis was performed to determine the minimum sample size for the study. Considering a type I error of 0.05 and 95% power, with a change of 1 mm in probing depth as the main clinical outcome of the study between the three study groups, based on a previous clinical study [15], the minimum sample size per group was 26 patients.

### 2.4. Periodontal Clinical Measurements

After the first session, subjects were randomly divided into three groups, using a computer-generated table: the group with conventional SRP and photoactivation therapy (PDT Group) (*n* = 50), the group with SRP and chlorhexidine irrigation 0.2% (CHX Group) (*n* = 67), and the group of subjects who followed only conventional mechanical therapy—SRP only (Control group) (*n* = 52). Nine subjects required the discontinuation of chlorhexidine rinsing (4 of them reported dental and mucosal pigmentation, 4 reported taste disorders, and a patient reported burning sensation at the mucosal level after rinsing with CHX), thus leading to the exclusion from the study.

Patients underwent a complex periodontal clinical examination, determining the following periodontal parameters: probing depth (PD), loss of clinical periodontal attachment (CAL), probing bleeding index (BOP), and bacterial plaque index, with the determination of periodontal diagnosis [16,17]. For the diagnosis of periodontitis, we took into consideration the staging system according to the new classification of periodontal diseases (Severity stages 1–4) [5].

The periodontal probing was performed with a manual periodontal probe (CP-12, Hu-Friedy Mfg. Co., LLC, Chicago, IL, USA) by two calibrated periodontists. Probing depth, together with the loss of clinical periodontal attachment, was measured at six points per tooth: mesial-vestibular, central-vestibular, distal-vestibular, mesial-oral, central-oral, distal-oral. The probing depth, i.e., the distance from the gingival margin to the base of the periodontal sulcus/pocket, was measured using a graduated periodontal probe with a standardized tip diameter of approximately 0.5 mm. The applied probe force was standardized to 0.25 *n*. Bleeding on probing index (BOP) was assessed as present if bleeding was evident within 30 s of probing or absent, if no bleeding was observed; the plaque index was also calculated as a percentage, calculating the ratio between the number of dental surfaces that had bacterial plaque to the total number of surfaces examined. Periodontal parameters were evaluated at baseline, one month, and six months after the first session.

All examiners were experienced periodontists and were calibrated by the principal investigator (MD). Calibration was accepted if the measurements of the corresponding sites were equal to ≥90%. Examinations were performed blindly to the method of therapy used in the individual patient.

### 2.5. Therapeutic Interventions

All patients underwent conventional manual non-surgical scaling and root planing (SRP) treatment in the first session. In the photoactivation study group, after careful removal of the granulation tissue and mechanical debridement of the tooth surface, the treatment was performed using PDT laser (HELBO, Photodynamic Systems GmbH, Wels, Austria). The photosensitizer, phenothiazine chloride (HELBO, Bredent medical GmbH & Co. KG), was applied to the dental surface and photoactivated for 30 s/site, on a wavelength of 660 nm and irradiation of 100 mW [18]. The photoactivation sessions were performed at baseline, at 7 and 14 days from baseline. 

Patients with conventional prosthetic treatments in the group with CHX followed, in addition to conventional therapy in the first session, oral rinses at home with 0.2% chlorhexidine solution, twice a day, for 14 days. All patients were instructed to follow oral hygiene measures (dental brushing with manual toothbrush and cosmetic toothpaste), and for subjects in the PDT and control groups, it was recommended not to use mouthwash. Any side effects or side effects related to photoactivation, CHX irrigation, or even conventional mechanical therapy have been closely monitored.

All periodontal treatments were performed by periodontal specialists blinded to the purpose of the study.

### 2.6. Microbiological Examination

Bacterial plaque samples were taken at baseline, one month and six months after the first session; the deepest periodontal pocket was selected from each patient for sampling. At the selected site, each tooth was isolated with sterile cotton rolls, the supra-gingival plaque was carefully removed, and the site was gently dried with air spray. For the collection of crevicular fluid, a sterile paper cone, size 35 (Dentsply Maillefer, Ballaigues, Switzerland) was inserted into the site and held in place for 30 s; then, it was immediately transferred to a sterile Eppendorf tube with transport medium (Medizone, Dolj, Romania).

#### 2.6.1. DNA Extraction

DNA extraction was performed with a commercial Wizard^®^ Genomic Purification Kit (Promega, Madison, Wisconsin, USA) following the manufacturer’s instructions. Simultaneously with the realization of the extraction protocol, control of contamination between samples was performed by processing a test sample represented by water with a degree of purity for molecular biology. The quantification and purity of the isolated DNA was determined by spectrophotometry using NanoPhotometer^®^ (Implen Gmbh, München, Germany). 

#### 2.6.2. Quantification of Periodontopathogens

The sequence of primers and probes used for qPCR counting of the periodontids studied is shown in Table 1. 

Periodontopagenic quantification was performed by real-time quantitative PCR, TaqMan method, using the Mx3005P qPCR platform (Stratagene, La Jolla, CA, USA). The amplification was performed after the following thermal program: initial denaturation at 95 °C for 10 min and 40 cycles of 95 °C-30 s, 60 °C-1 min. The qPCR reactions were performed in a total volume of 25 µL of which 2 µL of the DNA was isolated from the test sample, as well as 12.5 µL of GoTaq^®^ qPCR Master Mix solution, and 0.4 µL ROX; the volume of primer, probe, and biopure water were optimized to determine the effective concentration. In the case of *A. actinomycetemcomitans*, the concentration of primers and probes was 100 nM and 200 nM, respectively, for *P. gingivalis* 300 nM and 200 nM, *P. intermedia* 100 nM and 100 nM, *T. denticola* 300 nM and 100 nM, and in the case of *T. forsythia*, 100 nM and 100 nM.

### 2.7. Statistical Analysis

Clinical and microbiological measurements were expressed as percentages or median values (minimum, maximum). The Mann–Whitney U test was used for the statistical analysis of differences between the treatment modalities. Changes between the evaluation time-points were calculated using the Wilcoxon signed-rank test. The significance level was considered for *p* = 0.05. All statistical and calibration calculations were performed using SPSS, Ver. 23 (SPSS Inc., Chicago, IL, USA).

## 3. Results

Table 2 summarizes the demographic and general characteristics of the study groups at baseline.

The analysis of the study was performed on the following groups:

- The group that underwent SRP therapy plus photoactivation therapy (PDT Group) (*n* = 50 total patients; number of sites = 318) consisted of 14 patients with stage 1 periodontitis (S1) (number of sites = 79), 16 patients with stage 2 periodontitis (S2) (number of sites = 97), 10 patients with stage 3 periodontitis (S3) (number of sites = 68), and 10 patients with stage 4 periodontitis (S4) (number of sites = 74).
- The group that underwent SRP therapy plus irrigation with chlorhexidine 0.2% (CHX Group) (*n* = 58 total patients; number of sites = 276) consisted of 18 patients with stage 1 periodontitis (S1) (number of sites = 75), 10 patients with stage 2 periodontitis (S2) (number of sites = 67), 16 patients with stage 3 periodontitis (S3) (number of sites = 71), and 14 patients with stage 4 periodontitis (S4) (number of sites = 63).
- The group with SRP only therapy (Control Group) (*n* = 52 total patients; number of sites = 256) consisted of 8 patients with periodontitis stage 1 (S1) (number of sites = 51), 14 patients with periodontitis stage 2 (S2) (number of sites = 74), 16 patients with periodontitis stage 3 (S3) (number of sites = 77), and 14 patients with periodontitis stage 4 (S4) (number of sites = 54) (Table 2).

Regarding the severity prevalence, 24.11% of the study sites presented superficial periodontal destruction (periodontitis stage 1), 28.00% moderate destruction (S2), 25.42% periodontitis stage 3, and 22.47% periodontitis stage 4.

### 3.1. Periodontal Clinical Parameters

Regarding periodontal parameters at baseline, all subjects with fixed prosthetic appliances on natural teeth and periodontitis showed plaque and bleeding on probing indices with a value close to 100%. Moreover, we did not notice differences in probing depth and loss of periodontal clinical attachment between the intervention groups by severity of periodontal lesions at baseline (Table 3).

Evaluation of plaque index and bleeding index one month after the first session revealed significant reductions for all three groups (*p* < 0.001), except for the bleeding index in patients in the control group with severe periodontitis (stages 3 and 4 of severity) (Table 3). At the 6-month evaluation, the plaque index was still significantly lower than baseline, but the bleeding index maintained a significant difference only for the PDT group (all stages of periodontitis severity) and for CHX group (stages 1 and 2 of severity); we observed a tendency for increased values for the other subjects (Table 3).

Following analysis of probing depth and loss of clinical attachment one month from baseline, we observed significant reductions in the PDT group (all stages), the CHX group (only subjects with superficial periodontitis, S1, and moderate, S2) and only for subjects with superficial periodontitis in the control group. Although lower than baseline, PD and CAL failed to reach a threshold of statistical significance in patients with severe periodontitis in the CHX group and moderate and severe periodontitis in the SRP only group (Table 3). Moreover, following comparisons between groups of values from one month, improvements in PD and CAL were more significant for the group that also underwent photoactivation therapy (*p* < 0.001). 

At the 6-month evaluation, we observed that significant decreases for probing depth and attachment loss were maintained only for the PDT and CHX subjects (stages 1 and 2 of periodontitis severity). Moreover, for the control group and CHX group (stages 3 and 4), the mean PD and CAL values were even higher than baseline, although without a significant difference (Table 3). 

### 3.2. Subgingival Pathogens 

Following analysis of bacterial load in patients with fixed prosthetic devices on natural teeth, we did not notice significant differences between groups at baseline. At the one-month evaluation, we observed a significant reduction in all patients with superficial/moderate periodontitis who underwent PDT and CHX therapy (stages 1 and 2) (Table 4, Table 5 and Table 6), with supplementary improvements for patients who underwent photoactivation therapy. Patients with severe periodontitis (stages 3 and 4) who followed SRP+CHX rinsing, as well as those in the control group, although they showed lower values at the one-month evaluation, did not reach the threshold of statistical significance (Table 5 and Table 6). 

Although at the six-month evaluation we observed an upward trend, compared to the one month evaluation, significant lower values when compared to baseline were maintained for the PDT subjects and CHX subjects (S1 and S2) (Table 4 and Table 5). Moreover, subjects with periodontitis severity stages 3 and 4 who followed SRP only exhibited significant higher values than baseline (Table 6). 

In the study groups, *Aggregatibacter actinomycetemcomitans* was detected in subjects at baseline only in S2, S3, and S4 periodontal stages, the incidence being higher as the severity of periodontal disease increased. After one month, we observed a complete eradication for the pathogen in subjects with periodontitis stage 2 who followed PDT therapy, and the value was maintained after six months. CHX rinsing exerted a complete eradication for subjects with superficial periodontitis and significant lower incidence for subjects with periodontitis stage 2 and 3, but at 6 months, the incidence was close to baseline in subjects with periodontitis S2, S3, and S4 (Table 7, Table 8 and Table 9). PDT succeeded also in a significant reduction of *A. actinomycetemcomitans* in subjects with severe periodontitis, and, more importantly, the significant threshold of *p* < 0.05 was maintained also after 6 months. In the control group, a slight decrease was observed after one month only in patients with periodontitis stage 1 and 3 (Table 9). 

*Porphyromonas gingivalis* was detected in all groups, with higher prevalence for patients with severe periodontitis. Photoactivation therapy has resulted in the complete eradication in patients with stage 1 and 2 periodontitis after one month and significant decreases for subjects with severe periodontitis (S3, S4); the significant difference was observed also at the 6-month evaluation (Table 7). Irrigation with chlorhexidine 0.2% resulted in a significant reduction of *P gingivalis* in all the patients after one month, but after 6 months, the values increased again, with a tendency to return to baseline levels (Table 8). Conventional mechanical therapy resulted in a significant reduction in *P. gingivalis* only in patients with stage 1 and 2 periodontitis; however, at the 6-month evaluation, a trend of higher values for all stages of periodontitis severity was observed, similar to the CHX group (Table 9).

The complete eradication of *Tannerella forsythia* after one month was observed only in subjects with superficial periodontitis who underwent adjunctive photoactivation therapy. We observed significant results in decreased values in all the subjects with PDT after one month; after 6 months, the values slightly increased, but the difference maintained the statistical significance (Table 7). CHX rinsing generated similar results for patients with superficial periodontitis (S1) after one and six months, but even if the results were also significant for S2 patients at the first re-evaluation, after 6 months, the incidence increased, neutralizing the statistical significance; meanwhile, in patients with S3 and S4 periodontitis, even if CHX decreased the values, the difference was not significant (Table 8). Patients who followed only scaling and root planing exhibited decreases in *T. forsythia* incidence, but the difference was significant only for S1 patients after one month; at the 6-month evaluation, the values increased, reaching the baseline incidence (Table 9).

*Treponema denticola* was also detected in all the study groups. After one month, the photoactivation therapy resulted in significant reductions of this microorganism for all subjects (Table 7). CHX irrigation therapy also exerted significant reductions in *T. denticola* for S1 and S2 subjects; however, the results were significantly more favorable for photoactivation therapy (Table 8). Scaling and root planing resulted in significant reductions only in patients with superficial and moderate periodontitis (S1 and S2) (Table 9).

After 6 months, only PDT succeeded in maintaining the statistical significant decrease; in the CHX and control group, all the subjects experienced increased values of *T. denticola* incidence, with values close to baseline (Table 7, Table 8 and Table 9).

## 4. Discussion

Considering the fact that patients with fixed partial dentures have a worse periodontal status when compared to patients with natural dentition, periodontal maintenance and treatment must be carefully considered [19].

The present study proposed an investigation of the effects exerted on clinical and microbiological levels by photoactivation therapy as an additional periodontal therapeutic method in patients with prosthetic treatment on natural teeth, compared to additional irrigation with chlorhexidine 0.2% or single periodontal mechanical debridement. Although toluidine blue has generally been selected as a photosensitizer of choice in in vitro studies, methylene blue has been used primarily in clinical trials because clinical photodynamic therapy kits that include methylene blue are already commercially available (PeriowaveTM; Ondine Biopharma Corporation, Vancouver, Canada) (Helbo; Photodynamic Systems GmbH & Co. KG, Grieskirchen, Austria). Non-laser light sources, such as light-emitting diodes (LEDs), have been suggested as light activators in photodynamic therapy because LED devices are more compact and portable, and they cost much less than traditional lasers. In the present study, we used the laser as a light source (HELBO, Photodynamic Systems GmbH, Wels, Austria), with a wavelength of 660 nm and irradiation of 100 mW, and the photosensitizer was represented by phenothiazine chloride (HELBO, Bredent medical GmbH & Co. KG). The photosensitizer is placed directly in the periodontal pocket, and the liquid agent can easily access the entire root before activating the laser light by placing the optical fiber directly in site.

A previous study [20] compared the efficacy of antimicrobial photodynamic therapy with that of SRP for the non-surgical treatment of moderate to advanced periodontal disease. A total of 33 patients were assigned for single photodynamic therapy (methylene blue + 50 mW diode laser), single SRP, or SRP + photodynamic therapy. Clinical assessments of bleeding on probing, probing depth, and level of clinical attachment were performed. After three months, it was observed that a combination of SRP + photodynamic therapy led to significant improvements in the investigated parameters. Previous research evaluated the effect of adjunct antimicrobial photodynamic therapy (methylene blue + 100 mW diode laser) in chronic periodontitis using a split-mouth design. Twenty patients underwent an SRP procedure, and the arches were randomly assigned to an additional treatment with photodynamic therapy. After 3 months, complementary use of photodynamic therapy resulted in a significantly greater change in mean relative attachment level, probing depth, crevicular fluid flow, and bleeding on probing at sites that received photodynamic therapy compared to sites that followed only SRP [21]. 

On the other hand, other studies find no additional benefits to PDT as an adjunct to scaling and root planing regarding the reduction in pocket depth, bleeding on probing, clinical attachment depth, and eradication of bacteria when compared to conventional non-surgical treatment alone [22,23], while others find some benefits regarding bacteria recurrence and bleeding on probing [24]. These differences may be explained by the number of PDT applications, as it is highly likely that only one application is not sufficient to induce a significant difference in the clinical or microbiological parameters.

Wavelengths of 632.8 nm (helium-neon laser) and 665 nm and 830 nm (diode laser) have been shown to have a high bactericidal effect on periodontal pathogenic microorganisms [25]. In addition, the bactericidal effects of photodynamic therapy against the biofilm of the subgingival plaque, which included both Gram-positive and Gram-negative bacteria, were demonstrated; bacteria present in the deep layers of the biofilm were killed by the intense penetration of the photosensitizer into the biofilm [26]. In black pigment bacteria, such as *P. gingivalis* and *Prevotella spp*., endogenous porphyrins present on bacteria can also act as a photosensitizer. Moreover, it seems that antimicrobial photodynamic therapy not only kills bacteria, but it can also lead to a decrease in endotoxins; an in vitro study showed that lipopolysaccharides, following photodynamic therapy, did not stimulate the production of pro-inflammatory cytokines by mononuclear cells [27]; thus, photodynamic therapy can inactivate endotoxins by reducing their biological activity.

Yilmaz et al. [28] conducted a study in which they randomly assigned a number of ten patients to follow SRP + photodynamic therapy (methylene blue + 30 mW diode laser), single SRP, single photodynamic therapy, or supra-gingival oral hygiene instructions. Methylene blue served as a photosensitizer and was used to rinse the mouth. SRP was performed on days 1 and 7, while the laser was repeatedly applied to each papillary region (not in periodontal pockets) on days 1, 2, 4, 7, 9, and 11. After 32 days of healing, clinically significant and microbiological improvements were observed only in groups with SRP + photodynamic therapy and SRP only. On the contrary, the improvements in patients undergoing only photodynamic treatment, as well as those receiving oral hygiene instructions, did not reach statistical significance. Regarding laser treatment, there were no complaints (such as discomfort, tenderness or pain) from subjects immediately after or 3 weeks after therapy, which were aspects that were observed in our study as well. The authors concluded that antimicrobial photodynamic therapy did not provide additional microbiological and clinical benefits compared to conventional mechanical debridement. The reduced efficacy of photodynamic therapy in this study may be the result of indirect application of photodynamic therapy to the external surface of the gingiva. 

In our study, the photoactivation therapy with a photosensitizer was applied in the periodontal pocket, in three sessions, at an interval of one week apart, which resulted in significant improvements of clinical periodontal parameters in subjects with different severities of periodontal destruction. More interestingly, the improvements persisted even after 6 months since the first PDT session, which were beneficial results that were not observed in the chlorhexidine rinsing or SRP groups, especially in severe cases of periodontitis. On the other hand, regarding the bacterial plaque index, even if values at the one-month mark were significantly lower than baseline in the same study group, and the significance was maintained at 6 months, patients who rinsed with chlorhexidine and control subjects presented a tendency to increased plaque deposits and thus poor oral hygene, having higher overall scores, which were significant when compared to the photoactivation group. This particular aspect could partially explain the inter-group differences in terms of bacterial load and pathogen prevalence and the improved status for PDT treated subjects.

Moreover, regarding the microbiological profile, we demonstrated that PDT generated significant decreases in total bacterial load and specific periodontopathogenic bacteria, with a higher level of significance than additional chlorhexidine rinses or SRP alone. Importantly, the significant improvement was observed also after 6 months from baseline, proving the potential higher efficiency of this particular adjunctive method compared to CHX rinsing. 

The re-contamination of previous negative sites from deep periodontal pockets could partially explain the higher incidence of periodontal pathogens at the six-month evaluation. Furthermore, even if the basic oral hygiene measures of toothbrushing were explained to patients and re-instated at each session and the technology of oral hygiene self-control devices is increasing in evolution, there is still no accurate method to assure that these practices were correctly followed; this particular aspect of patient motivation continues to represent a challenge in dental practice.

Chlorhexidine is a cationic polybiguanide (bisbiguanide). It is an antibacterial agent and is used as an antiseptic for other applications. Chlorhexidine is used in disinfectants, cosmetics, and pharmaceuticals. At physiological pH, the chlorhexidine salts dissociate and release the positively charged chlorhexidine cation. The bactericidal effect is the result of the binding of this cationic molecule to the walls of negatively charged bacterial cells. At low concentrations of chlorhexidine, a bacteriostatic effect results; at high concentrations, membrane disruption results in cell death [29]. For dental plaque and inflammation control, chlorhexidine is used for irrigation or in oral rinses in its digluconate form. Due to its very high therapeutic index, it is one of the most widely used antiseptics today. In the present study, additional rinses with chlorhexidine 0.2%, twice daily, for 14 days, generated satisfactory clinical and microbiological results, but in general, they were limited to patients with superficial and moderate forms of periodontitis; these aspects might suggest that CHX is ineffective in reaching deep periodontal pockets. Furthermore, it has been reported that antibiotics and antiseptics can lead to bacterial resistance and unwanted side effects [30], which are effects that are also found in the present study that led to the elimination of nine subjects from the study. These limitations have led to the search for new approaches that are effective and easy to apply in periodontal disease.

PDT efficacy has been reported to be greater in controlling or eliminating oral bacteria in the planktonic phase than in biofilms [31]. Probable reasons for the low effects of PDT in biotopes may be distinct and protected phenotypes, such as those of microorganisms in dental bacterial plaques that are able to adhere to teeth [32]. To this end, scaling and root planing therapy for the disorganization of the bacterial biofilm remains an essential step, irreplaceable, in the periodontal treatment. Nevertheless, we cannot ignore the limitations of this procedure in deep periodontal pockets of severe periodontitis patients, where access might be impeded; in these particular situations, open flap debridement is generally recommended. However, the results of our study prove the efficiency of PDT therapy even in profound periodontal pockets, thus suggesting the possibility of reducing the need for surgical periodontal procedures.

Since antimicrobial photodynamic therapy can be applied topically, systemic administration of antibiotics can be avoided in the treatment of localized infections. In antimicrobial photodynamic therapy, a high concentration of the chemical agent at the infection site allows efficient elimination of bacteria, without generating side effects on the patient’s tissues [33]. Several photodynamic therapy sessions can improve healing results and its long-term effects. However, it has not been established how often photodynamic therapy should be applied to effectively eliminate bacteria, as well as to prevent the recolonization by bacteria of sites previously treated by non-surgical periodontal therapy. Another adjunctive method that has been proposed recently in the literature is the administration of probiotics, either systemically or locally [34], and further studies should compare the two adjunctive methods for efficacy assessment. 

As limitations of the study, we acknowledge the fact that the evaluations in the present study were performed one and six months after the first session; thus, supplementary research is needed to elucidate whether multiple sessions of antimicrobial photodynamic therapy can improve treatment outcomes in the long term using more complex statistical tools such as correlation matrix analysis. Furthermore, all subjects in our study followed manual scaling and root planing; studies on motor-driven instruments and their antibacterial efficiency compared to PDT are also necessary. Regarding the microbiological determinations, in our study, we analyzed only four periodontopathogens: *Aggregatibacter actinomycetemcomitans, Porphyromonas gingivalis, Tannerella forsythia,* and *Treponema denticola*; however, a more complete microbiological analysis is necessary for the bacterial profiling of these patients, before and after periodontal treatment.

## 5. Conclusions

Adjunctive photoactivation antimicrobial therapy resulted in significantly more important clinical and microbiological results, especially in cases of periodontitis with severe tissue loss, when compared to SRP alone or SRP plus chlorhexidine therapy, without any side effects. Oral rinses with chlorhexidine 0.2% generated favorable clinical and microbiological results, particularly in patients with conventional fixed prosthetic treatment with superficial and moderate periodontitis but also caused adverse reactions.

Antimicrobial photodynamic therapy is expected to solve the difficulties and problems of conventional antimicrobial therapy and can function as an adjunct to conventional mechanical treatments.

## Figures and Tables

**Table 1:** Sequence of primers and probes.

Patogen	Primer 5′→3′	Probe 5′→3′	Gene
*A. a*	F: GCGAACGTTAGCGTTTTAC R: GGCAAATAAACGTGGGTGAC	ATTGCCCGCACCGAAACCCAAC 5′_Cy5→BHQ2_3′	waaA
*P. g*	F: TGGTTTCATGCAGCTTCTT R: TCGGCACCTTCGTAATTCTT	GTACCTCATATCCCGAGGGGCTG 5′_HEX→BHQ1_3′	waaA
*T. d*	F: CCTTGAACAAAAACCGGAA R: GGGAAAAGCAGGAAGCATAA	GAGCTCTGAATAATTTTGATGCA 5′_Cy5→BHQ2_3′	waaG
*T. f*	F: CTCGCTCGGTGAGTTTGAA R: ATGGCGAAAAGAACGTCAAC	CGATTCGCAAGCGTTATCCCGACT 5′_HEX→BHQ1_3′	waaA

*A. a = Aggregatibacter actinomycetemcomitan; P. g = Porphyromonas gingivalis; T. d = Treponema denticola; T. f = Tannerella forsythia*.

**Table 2:** General characteristics of study subjects.

Group	Periodontitis Severity	Number of Subjects	Sites *n* (%)	Age (Years) (Mean ± SD)	Gender (%)	
					Male	Female
PDT	S1	14	79 (9.29%)	47.3 ± 7.4	42.8	57.2
	S2	16	97 (11.41%)	52.5 ± 10.2	50.5	49.5
	S3	10	68 (8.00%)	53.1 ± 10.1	60.0	40
	S4	10	74 (8.71%)	47.9 ± 9.6	75.0	25
CHX	S1	18	75 (8.82%)	45.3 ± 7.9	66.7	33.3
	S2	10	67 (7.88%)	47.5 ± 7.6	60.0	40
	S3	16	71 (8.35%)	57.2 ± 11.9	62.5	37.5
	S4	14	63 (7.41%)	50.3 ± 12.4	71.4	28.6
Control	S1	8	51 (6.00%)	46.9 ± 8.8	50.0	50.0
	S2	14	74 (8.71%)	52.1 ± 10.8	42.8	57.2
	S3	16	77 (9.07%)	50.8 ± 11.4	50.0	50
	S4	14	54 (6.35%)	49.2 ± 8.6	57.1	42.9

PDT: SRP + photoactivation therapy group; CHX: SRP + chlorhexidine rinsing group; SD: standard deviation. S1: Periodontitis stage 1; S2: Periodontitis stage 2; S3: Periodontitis stage 3; S4: Periodontitis stage 4.

**Table 3:** Periodontal parameters on study groups and periodontitis severity at baseline, one month and six months evaluation.

Parameter	PDT				CHX				Control			
	S1	S2	S3	S4	S1	S2	S3	S4	S1	S2	S3	S4
Baseline evaluation												
PI (+)(%)	98.7	97.9	95.6	92.6	98.7	94.0	95.8	90.5	96.0	97.3	92.2	92.6
BOP (+)(%)	87.3	85.6	89.7	93.2	89.3	88.1	87.3	85.7	86.3	91.9	91.0	87.0
PD (Mean ±DS)	3.24± 0.42	4.40± 0.21	5.21± 1.93	7.20 ± 1.18	3.21± 0.74	4.20± 0.95	5.18± 1.27	7.50± 1.72	3.01± 0.79	4.10 ± 1.13	5.15 ± 1.87	6.90 ± 1.41
CAL (Mean± DS)	1.40 ± 0.32	3.50 ± 0.44	4.22 ± 0.42	5.68 ± 1.16	1.61 ± 0.28	3.23 ± 0.33	4.48 ± 0.99	5.90 ± 1.24	1.42 ± 0.78	3.19 ± 0.32	4.21 ± 1.31	5.41 ± 1.75
One month evaluation												
PI (+)(%)	5.1 *	6.2 *	3.0 *	8.1 *	1.3 *	7.5 *	11.2 *	12.7 *	4.0 *	8.1 *	7.8 *	7.4 *
BOP (+)(%)	2.5 *	2.1 *	1.5 *	4.1 *	4.0 *	3.0 *	5.7 *	9.5 *	13.7 *	12.2 *	31.6	42.2
PD (Mean± SD)	1.98 ± 0.31 *	2.21 ± 0.22 *	3.87 ± 0.41 *	5.42 ± 0.84 *	1.87 ± 0.63 *	3.12 ± 0.32 *	4.99 ± 0.55	6.62 ± 0.41	1.95 ± 0.1 *	3.72 ± 0.88	5.01 ± 1.16	6.39 ± 1.23
CAL (Mean± SD)	1.12 ± 0.42 *	1.81 ± 0.21 *	2.03 ± 0.35 *	4.32 ± 1.63 *	1.13 ± 0.17 *	2.67 ± 0.43 *	4.21 ± 1.17	5.22 ± 1.73	0.82 ± 0.24 *	2.93 ± 0.52	4.19 ± 1.16	5.40 ± 1.97
Six months evaluation												
PI (+)(%)	4.2 *	4.7 *	2.6 *	7.2 *	3.6 *	9.6 *	12.5 *	14.2 *	15.3 *	14.9 *	16.5 *	17.2 *
BOP (+)(%)	1.3 *	1.7 *	1.2 *	2.5 *	23.7 *	26.9 *	43.6	61.7	39.42	38.3	42.8	57.1
PD (Mean± SD)	1.61 ± 0.37 *	1.82 ± 1.41 *	3.34 ± 1.66 *	5.23 ± 1.92 *	2.02 ± 1.14 *	3.51 ± 1.42 *	5.29 ± 1.90	6.97 ± 2.46	3.05 ± 1.53	4.61 ± 2.07	5.56 ± 2.44	7.19 ± 2.88
CAL (Mean± SD)	0.95 ± 0.14 *	1.34 ± 0.27 *	1.88 ± 0.41 *	4.75 ± 2.63 *	1.27 ± 0.43 *	2.93 ± 0.63 *	4.65 ± 1.04	5.98 ± 1.48	1.24 ± 0.41	3.46 ± 0.73	4.91 ± 1.77	6.22 ± 2.24

PDT: SRP + photoactivation therapy group; CHX: SRP + chlorhexidine rinsing group; SD: standard deviation. S1: Periodontitis stage 1; S2: Periodontitis stage 2; S3: Periodontitis stage 3; S4: Periodontitis stage 4. PI: Plaque index; BOP: Bleeding on probing index; PD: periodontal probing depth; CAL: clinical attachment loss; SD: standard deviation. * *p* < 0.05 was considered statistically significant.

**Table 4:** Total bacterial load at baseline and at after treatment (one month and six months) in the PDT group.

Stage		Baseline	At One Month		At 6 Months
S1	5.4 × 10^6^ (2.6 × 10^4^–8.3 × 10^8^)	1.6 × 10^3^ (9.5 × 10^2^–8.9 × 10^4^) *		5.2 × 10^3^ (1.2 × 10^2^–7.5 × 10^5^) *	
S2	1.6 × 10^7^ (3.8 × 10^6^–5.2 × 10^9^)	9.6 × 10^3^ (6.2 × 10^2^–9.2 × 10^4^) *		1.7 × 10^4^ (9.1 × 10^2^–9.6 × 10^5^) *	
S3	3.2 × 10^8^ (6.8 × 10^6^–9.8 × 10^9^)	6.9 × 10^4^ (1.4 × 10^3^–8.1 × 10^7^) *		1.4 × 10^5^ (2.7 × 10^3^–8.3 × 10^7^) *	
S4	8.3 × 10^8^ (5.2 × 10^6^–9.3 × 10^9^)	8.5 × 10^4^ (2.5 × 10^3^–1.2 × 10^7^) *		1.8 × 10^5^ (5.5 × 10^3^–5.3 × 10^7^) *	

PDT: SRP + photoactivation therapy group; S1: Periodontitis stage 1; S2: Periodontitis stage 2; S3: Periodontitis stage 3; S4: Periodontitis stage 4. Data are expressed as median (minimum-maximum). * *p* < 0.05; comparison between the evaluation at baseline and at one and 6 months in the same group.

**Table 5:** Total bacterial load at baseline and at after treatment (one month and six months) in CHX group.

Stage	Baseline	At One Month	At 6 Months
S1	5.6 × 10^6^ (6.2 × 10^4^–7.5 × 10^8^)	8.4 × 10^4^ (5.5 × 10^2^–2.1 × 10^6^) *	3.7 × 10^5^ (9.1 × 10^2^–9.2 × 10^6^) *°
S2	1.9 × 10^7^ (4.6 × 10^6^–9.2 × 10^9^)	0.5 × 10^4^ (7.8 × 10^2^–6.3 × 10^6^) *	6.2 × 10^5^ (9.3 × 10^2^–8.7 × 10^6^) *°
S3	3.6 × 10^8^ (6.5 × 10^6^–9.1 × 10^9^)	9.1 × 10^7^ (2.9 × 10^3^–0.1 × 10^9^) °	1.8 × 10^8^ (7.4 × 10^5^–7.5 × 10^9^) °
S4	7.9 × 10^8^ (2.9 × 10^6^–8.3 × 10^9^)	0.3 × 10^8^ (1.8 × 10^5^–2.3 × 10^9^) °	6.2 × 10^8^ (5.9 × 10^5^–8.4 × 10^9^) °

CHX: SRP + chlorhexidine rinsing group. S1: Periodontitis stage 1; S2: Periodontitis stage 2; S3: Periodontitis stage 3; S4: Periodontitis stage 4. Data are expressed as median (minimum–maximum). * *p* < 0.05; comparison between the evaluation at baseline and at one and 6 months in the same group; ° *p* < 0.05; comparison between groups at the same evaluation time.

**Table 6:** Total bacterial load at baseline and at after treatment (one month and six months) in control group (SRP only).

Stage	Baseline	At One Month	At 6 Months
S1	4.9 × 10^6^ (1.6 × 10^4^–2.3 × 10^8^)	0.3 × 10^6^ (1.5 × 10^4^–1.2 × 10^8^) °	5.1 × 10^6^ (5.5 × 10^4^–4.7 × 10^8^) °
S2	1.7 × 10^7^ (7.1 × 10^6^–8.1 × 10^8^)	0.2 × 10^7^ (1.5 × 10^6^–6.4 × 10^8^) °	2.3 × 10^7^ (5.9 × 10^6^–7.8 × 10^8^) °
S3	3.1 × 10^8^ (6.3 × 10^6^–8.8 × 10^9^)	2.1 × 10^8^ (5.2 × 10^6^–7.3 × 10^9^) °	2.7 × 10^9^ (7.9 × 10^7^–9.1 × 10^9^) †°
S4	7.8 × 10^8^ (6.5 × 10^6^–8.1 × 10^9^)	6.6 × 10^8^ (4.2 × 10^6^–5.3 × 10^9^) °	8.7 × 10^9^ (8.8 × 10^8^–9.8 × 10^9^) †°

S1: Periodontitis stage 1; S2: Periodontitis stage 2; S3: Periodontitis stage 3; S4: Periodontitis stage 4. Data are expressed as median (minimum-maximum). † *p* < 0.05; comparison between the evaluation at baseline and at one and 6 months in the same group with significant increase. ° *p* < 0.05; comparison between groups at the same evaluation time.

**Table 7:** Periodontal pathogens detection at baseline and after treatment (one month and six months) for the PDT group.

Pathogen	Stage	Baseline	At One Month	At 6 Months
*Aggregatibacter actinomycetemcomitans*	S1	0.0	0.0	0.0
	S2	2.1	0.0 ^a^	0.0 ^a^
	S3	30.9	1.4 ^b^	2.9 ^b^
	S4	33.8	2.7 ^b^	5.4 ^b^
*Porphyromonas gingivalis*	S1	6.3	0.0 ^b^	1.3 ^b^
	S2	14.4	0.0 ^b^	2.1 ^b^
	S3	63.2	4.4 ^b^	10.3 ^b^
	S4	83.8	6.7 ^b^	10.9 ^b^
*Tannerella forsythia*	S1	30.4	0.0 ^b^	2.5 ^b^
	S2	32.9	4.1 ^b^	6.2 ^b^
	S3	67.6	8.8 ^b^	10.3 ^b^
	S4	93.2	8.1 ^b^	12.2 ^b^
*Treponema denticola*	S1	22.8	2.5 ^b^	3.8 ^b^
	S2	29.9	2.1 ^b^	5.1 ^b^
	S3	57.4	5.8 ^b^	7.3 ^b^
	S4	66.2	8.1 ^b^	10.8 ^b^

PDT: photoactivation therapy group; S1: Periodontitis stage 1; S2: Periodontitis stage 2; S3: Periodontitis stage 3; S4: Periodontitis stage 4. Results are expressed as percentages. ^a^ *p* < 0.005; ^b^ *p* < 0.001; comparison between the evaluation at baseline and at one and 6 months in the same group.

**Table 8:** Periodontal pathogens detection at baseline and after treatment (one month and six months) for the CHX group.

Pathogen	Stage	Baseline	At One Month	At 6 Months
*Aggregatibacter actinomycetemcomitans*	S1	0.0	0.0	0.0
	S2	2.9	1.2	1.5
	S3	26.8	14.1 ^a^	23.9
	S4	30.1	19.1	22.2
*Porphyromonas gingivalis*	S1	9.3	4.0 ^c^	8.0
	S2	16.4	5.9 ^c^	13.4
	S3	63.4	30.9 ^c^	47.9
	S4	85.7	30.2 ^c^	71.8
*Tannerella forsythia*	S1	34.7	4.0 ^c^	14.7 ^b^
	S2	37.3	4.5 ^c^	32.8
	S3	57.7	32.4	47.9
	S4	85.7	69.8	73.0
*Treponema denticola*	S1	22.7	1.3 ^c^	21.6
	S2	32.8	4.5 ^c^	29.8
	S3	54.9	36.7	43.7
	S4	73.0	55.5	72.2

CHX: Chlorhexidine rinsing group; S1: Periodontitis stage 1; S2: Periodontitis stage 2; S3: Periodontitis stage 3; S4: Periodontitis stage 4. Results are expressed as percentages. ^a^ *p* < 0.05; ^b^ *p* < 0.005; ^c^ *p* < 0.001; comparison between the evaluation at baseline and at one and 6 months in the same group.

**Table 9:** Periodontal pathogens detection at baseline and after treatment (one month and six months) for the control group.

Pathogen	Stage	Baseline	At One Month	At 6 Months
*Aggregatibacter actinomycetemcomitans*	S1	0.0	0.0	0.0
	S2	4.1	2.7	2.7
	S3	28.6	22.1	24.7
	S4	35.2	29.6	31.5
*Porphyromonas gingivalis*	S1	9.8	3.9 ^b^	7.8
	S2	17.6	8.1 ^b^	13.5
	S3	62.3	45.5	51.9
	S4	81.4	66.7	74.1
*Tannerella forsythia*	S1	33.3	13.7 ^a^	27.4
	S2	39.2	27.0	29.7
	S3	76.6	57.1	67.3
	S4	90.7	77.8	85.9
*Treponema denticola*	S1	19.6	5.9 ^b^	17.7
	S2	28.4	14.9 ^a^	25.7
	S3	58.4	41.6	44.1
	S4	74.1	57.4	66.7

S1: Periodontitis stage 1; S2: Periodontitis stage 2; S3: Periodontitis stage 3; S4: Periodontitis stage 4. Results are expressed as percentages. ^a^ *p* < 0.005; ^b^ *p* < 0.001; comparison between the evaluation at baseline and at one and 6 months in the same group.

## Data Availability

The data used to support the findings of this study are available from the corresponding authors upon reasonable request.
